# Framework for Race-Specific Prostate Cancer Detection Using Machine Learning Through Gene Expression Data: Feature Selection Optimization Approach

**DOI:** 10.2196/72423

**Published:** 2025-07-31

**Authors:** David Agustriawan, Adithama Mulia, Marlinda Vasty Overbeek, Vincent Kurniawan, Jheno Syechlo, Moeljono Widjaja, Muhammad Imran Ahmad

**Affiliations:** 1 Faculty of Engineering and Informatics, Universitas Multimedia Nusantara, Scientia Garden Jalan Boulevard Gading Serpong, Tangerang, 15810, Indonesia, 62 877-8153-5936; 2 Faculty of Electronic Engineering and Technology, Universiti Malaysia Perlis, Perlis, Malaysia

**Keywords:** prostate cancer, feature selection, gene expression, race specific, classification, support vector machine, machine learning

## Abstract

**Background:**

Previous machine learning approaches for prostate cancer detection using gene expression data have shown remarkable classification accuracies. However, prior studies overlook the influence of racial diversity within the population and the importance of selecting outlier genes based on expression profiles.

**Objective:**

We aim to develop a classification method for diagnosing prostate cancer using gene expression in specific populations.

**Methods:**

This research uses differentially expressed gene analysis, receiver operating characteristic analysis, and MSigDB (Molecular Signature Database) verification as a feature selection framework to identify genes for constructing support vector machine models.

**Results:**

Among the models evaluated, the highest observed accuracy was achieved using 139 gene features without oversampling, resulting in 98% accuracy for White patients and 97% for African American patients, based on 388 training samples and 92 testing samples. Notably, another model achieved a similarly strong performance, with 97% accuracy for White patients and 95% for African American patients, using only 9 gene features. It was trained on 374 samples and tested on 138 samples.

**Conclusions:**

The findings identify a race-specific diagnosis method for prostate cancer detection using enhanced feature selection and machine learning. This approach emphasizes the potential for developing unbiased diagnostic tools in specific populations.

## Introduction

### Prostate Cancer Statistics

Prostate cancer is the most common type of organ cancer and the second leading cause of death in the United States among men [1,2]. In 2019, over 893,660 cancer cases were recorded in the United States, with prostate cancer being over 191,930 of them, along with the 2020 estimated number of deaths caused by cancer being 321,160, of which 33,310 were prostate cancer [3-5]. This is likely caused by risk factors found in prostate cancer that include age, family history, and lifestyle. Studies have shown that Asians tend to have a lower risk of prostate cancer than Europeans and Africans due to their genetics and environmental differences [6]. This indicates racial disparity in prostate cancer, which has been extensively documented by numerous studies, with African American men having a higher risk of developing prostate cancer and facing a 2.5-fold higher mortality rate compared to European American men [7,8]. This disparity is attributed to socioeconomic and biological differences, including aggressive tumor phenotypes documented at the molecular level in African American men [9].

### Prostate Cancer Detection Methods

In the early 1990s, digital rectal examination was used for screening prostate cancer, which had a significant impact on prostate cancer diagnosis at the time. Digital rectal examination remains beneficial for distinguishing between benign and malignant conditions in the prostate, but it is limited by its low sensitivity and inability to detect cancer at an early stage [3,10,11]. Another screening method is the prostate-specific antigen (PSA) test. While widely used, PSA testing is controversial due to its susceptibility to false positives, as PSA is a gland-specific biomarker rather than cancer-specific biomarker [10,12]. The lack of a reliable and robust detection method gives rise to the need for a race-based approach to detect prostate cancer.

### Machine Learning and Support Vector Machine

In recent years, machine learning applications in health care and biotechnology have grown rapidly, driving advancements in disease diagnostics, personalized medicine, and bioinformatics [13]. In this research, support vector machines (SVMs) were selected for their remarkable performance in classification tasks in the medical field using gene expression data [14-18]. Being a supervised machine learning algorithm that is proficient at distinguishing between 2 sample classes, SVM works by creating a hyperplane that optimally separates sample classes. SVM transforms class data into a higher-dimensional space to effectively identify complex, nonlinear relationships. This makes SVM especially powerful in cases with small sample sizes and high-dimensional data, such as gene expression profiles or genomic datasets. These characteristics made SVM an invaluable algorithm in bioinformatics, where the classification of diseases such as cancer requires robust, data-driven methods to handle variability and heterogeneity [10,15].

### Gene Expression Data

Gene expression is a process where information in DNA becomes instructions to make proteins or other molecules [16,19]. The process starts when DNA is copied into mRNA and changed into proteins. Gene expression analysis is typically used for monitoring genetic changes in tissues or single cells under certain conditions. It checks how many DNA transcripts are in a sample to know which genes are active and by how much, including comparing the sequenced reads with the number of base pairs from a DNA piece to a known genome or transcriptome. The process’ accuracy depends on the clarity of information obtained, which allows bioinformatics tools to match them to the right genes. However, the gene expression dataset poses an additional challenge due to their high dimensionality, where the ratio of features to samples is high, hindering the performance of classification models. To address this, researchers have used feature selection methods to filter out irrelevant or redundant genes [20,21]. Feature selection has a critical role in improving machine learning models’ classification outcomes in high-dimensional datasets, making it a basis for an efficient classification model for cancer detection [22,23].

### Racial Dataset Influence in Artificial Intelligence

Racial-based genomic datasets present challenges for machine learning applications. Studies have shown that using race-based genomics data for artificial intelligence algorithms may exhibit biases where trained models favor the majority race in training data, lowering the accuracy on the minority races [8,24]. Racial class imbalance in the dataset, where certain races have more samples, can influence the accuracy of algorithms. However, when the class imbalance is less severe, the algorithms tend to achieve higher balanced accuracy across all racial groups [25]. To mitigate this, an approach that reweighs the minority classes is performed, yet this approach was unreliable when the class imbalance is severe [24,26]. This research uses race-based genomics data instead of a combined race dataset to address the biases that may appear when using a combined dataset.

### Prior Research and Objective

Despite significant advancements in machine learning and prostate cancer diagnosis, a gap remains in addressing racial disparities in prostate cancer. A recent study by Alshareef et al [27] introduces artificial intelligence–based feature selection with deep learning model for prostate cancer detection, a newly developed method of prostate cancer detection using deep learning approach using microarray gene expression data with 52 prostate samples and 50 normal samples on 2135 genes [28]. It focuses on feature selection using Chaotic Invasive Weed Optimization and hyperparameter tuning over multiple iterations of the proposed artificial intelligence–based feature selection with deep learning model for prostate cancer detection model which leads to an average accuracy of 97.19%, precision of 97.14%, and *F*_1_-score of 97.28%. Similarly, Ravindran et al [29] proposed a prediction deep learning model for prostate cancer which focuses on data augmentation using the Wasserstein Tabular Generative Adversarial Network technique, which enables powerful discriminators that supply reliable gradient information to the sample generator even with poor sample qualities, allowing for a more stable training process [27]. The research uses a Micro Gene Expression Cancer Dataset (MGECD), of which the prostate cancer MGECD consists of 102 samples and 6033 features, and feature selection based on correlation coefficients with the goal of reducing the features to 1/3 of the initial MGECD by applying a threshold of 0.7. This results in 1833 features being used for the final model that has a 97% accuracy, 98% precision, and 97% recall values, a total of 3.4% accuracy improvement on prostate cancer classification using Wasserstein Tabular Generative Adversarial Network SVM compared to only using SVM. Previous research has demonstrated admirable results with limited amounts of samples, yet the proposed methods do not account for the racial biases that may be present in gene expression data and the number of genes needed to efficiently train machine learning models. To bridge this gap, we use feature selection methods such as differentially expressed gene (DEG) analysis, receiver operating characteristic (ROC) analysis, and MSigDB (Molecular Signature Database) verification. Our goal is to develop a race-based SVM model that improves prostate cancer detection for White populations and provides a novel genomics-based approach for health care professionals.

## Methods

### Study Design

**Figure 1.**
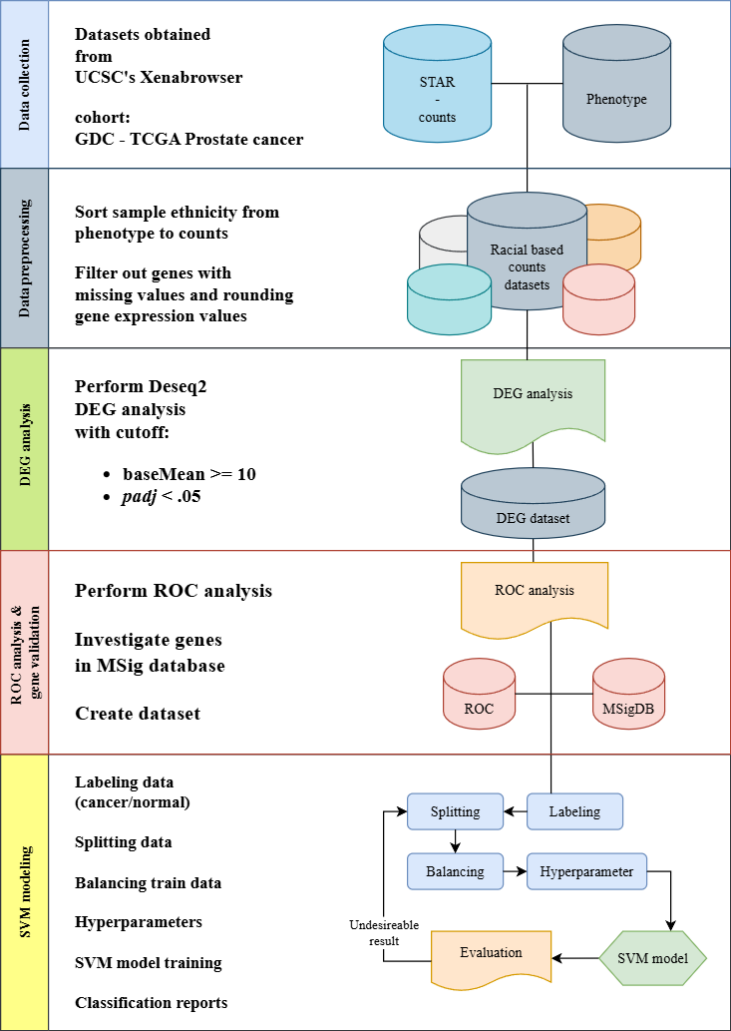
Race-specific prostate cancer detection modeling framework. DEG: differentially expressed gene; GDC: Genomic Data Commons; MSigDB: Molecular Signature Database; ROC: receiver operating characteristic; STAR: Spliced Transcripts Alignment to a Reference; SVM: support vector machine; TCGA: The Cancer Genome Atlas; UCSC: University of California, Santa Cruz.

This study implements data collection, preprocessing, feature selection, and SVM modeling and evaluation as seen in Figure 1. These methods are conducted using Python (version 3.12.3; Python Software Foundation) programming language and the necessary libraries using Visual Studio Code editor (version 1.95.3; Microsoft Corp) [30].

### Ethical Considerations

This study used publicly available datasets from the University of California, Santa Cruz Xena [31]. University of California, Santa Cruz Xena allows users to explore functional genomic data sets for correlations between genomic or phenotypic variables. Thus, no ethics approval was required.

### Data Collection

This study implements a structured methodology to identify and model significant genes for prostate cancer using gene expression data. There are 2 datasets used and obtained in August 2024 from Xenabrowser’s GDC (Genomic Data Commons) TCGA-PRAD (The Cancer Genome Atlas Prostate Adenocarcinoma) cohort, of which 1 contains gene expression counts data, and the other contains the clinical information of the samples [29]. Gene expression dataset has been prenormalized by Xenabrowser using log2(count+1).

### Data Preprocessing

Data preprocessing involved separating the counts dataset racially by mapping the samples to their race in the phenotype dataset, filtering samples with missing gene expression values, and labeling samples as normal or cancer via the TCGA (The Cancer Genome Atlas) barcode. These steps were conducted using the Pandas (version 2.2.2; NumFOCUS, Inc) and NumPy (version 1.26.4; NumPy Developers) libraries in a Jupyter Notebook (LF Charities) environment [32-34].

### Feature Selection

Feature selection to train the machine learning model was achieved through refining the filtered genes from DEG analysis, performed using the *PyDESeq2* package (version 0.4.10; OWKIN) [35-37]. After creating metadata and the appropriate data frame, we used the DESeqDataSet function to create a suitable dataset for the DESeq2 process. There are 3 parameters used in creating the DESeqDataSet. First is counts, which is where the data frame of gene expression values of each gene ID and sample ID is used. To create metadata for the DeseqDataSet function, we specify the design of the DEG experiment and the factors to be analyzed. The factors in this research are labeled sample IDs with their condition that has been converted to a data frame by using the DeseqStats function. Lastly, we defined the design factor to guide the DEG analysis to focus on the important variables, in this case, the sample conditions. Identifying significant genes is based on the set threshold of baseMean≥10 and *p-adj*<.05. The filtered genes were used to create 5 experimental scenarios, with the first scenario focusing on the outlier genes identified through *PyDESeq2* that met the specified thresholds.

The second and third scenarios were developed by introducing additional thresholds to the DEG results. The additional scenarios further narrowed down the outlier genes by applying log2FoldChange>0.35 and >0.4, respectively.

For the fourth scenario, ROC analysis was performed using the scikit-learn metrics library (version 1.5.1; scikit-learn developers) to isolate genes with high predictive impact [38,39]. Genes were filtered based on a cutoff threshold of area under the curve value above 0.90, and the results were visualized using the matplotlib library (version 3.9.1; The Matplotlib development team) [40]. These genes were then used to create the fourth scenario.

The final scenario involves converting the isolated genes’ Ensembl IDs into gene symbols using BioTools.fr for the human species Ensembl format [41] and verifying using gene set enrichment analysis (GSEA). Gene symbols were queried to MSigDB from GSEA to compute overlaps on curated gene sets which enables identification of well-established biological pathways and is widely used in cancer immunology and metabolic research, computational gene sets to complement the curated gene sets by providing unbiased large-scale insights and specific gene expression patterns, oncogenic gene sets that are directly relevant to cancer research and linked to gene expression changes on specific oncogenic events, and False Discovery Rate q-value less than 0.05 to reduce the likelihood of false positives in enrichment results [22,42-46]. Overlaps between the queried genes and the gene sets in MSigDB were analyzed to validate their relevance to prostate cancer. Genes with confirmed prostate cancer relevance were selected for use in the final scenario.

### SVM Modeling

The dataset initially shows a strong class imbalance, with a cancer-to-normal ratio of 1:9. To address this class imbalance, the data were split into training and testing sets using various stratified splits: 60%/40%, 70%/30%, and 80%/20%. Stratification ensures that the class distribution among the training data class imbalance was then addressed on all the training data scenarios using oversampling methods, including RandomOverSampler, SVMSMOTE, SMOTEENN, SMOTETomek, ADASYN, BorderlineSMOTE, and KMeansSMOTE from sci-kit libraries with a sampling strategy of 0.3, meaning the training data consists of 66.66% cancer samples and 33.33% normal samples, creating a balanced dataset for model training and preserving the authenticity of the testing data, making a realistic environment for the model to perform in.

Multimedia Appendix 1 (Table S1) and Table 1 show multiple experimental scenarios that were designed to test different parameter combinations and datasets. Two modeling scenarios were used; first, using the default SVC function with linear kernel. Second, conducting hyperparameter tuning to optimize model performance. Hyperparameter tuning was performed using GridSearchCV with a linear kernel SVC classifier and 5-fold cross-validation. The hyperparameters and their ranges were as follows: multiple kernels of the SVC function were used, linear, polynomial, and radial basis function. C values were ranging from 0.01, 0.1, 1, and 10, with gamma values of 0.01, 0.1, and 1, coef0 values of 0 and 1, and lastly class weights of none and balanced.

Evaluation of the model was obtained and inspected using the *classification_report* function, by focusing on harmonization between *F*_1_-score, recall, accuracy, precision, and macro-avg values, we evaluated the models’ performance on training and test sets to ensure reliability of the model with no over- or underfitting present. To further validate the results of the obtained machine learning model, we tested the model on a black dataset with corresponding gene amounts to further investigate the racial differences in prostate cancer. This approach aligns with the goal of improving the identification of prostate cancer within a specific population.

**Table 1.** Top 5 models for 4-gene scenario.

Balancing method	Data splitting ratio	Hyper-parameter	White					Black			
			Train accuracy (%)	Test accuracy (%)	*F*_1_-score (%)	Precision (%)	Recall (%)	Test accuracy (%)	*F*_1_-score (%)	Precision (%)	Recall (%)
KMeansSMOTE	80:20	Yes	94.2	94.6	97	94.3	100	93.7	96.5	94.9	98.2
KMeansSMOTE	70:30	No	92.8	93.5	96.5	94.6	98.4	93.7	96.5	94.9	98.2
KMeansSMOTE	80:20	No	92.8	93.5	96.4	95.3	97.6	92.2	95.6	94.8	96.5
SVMSMOTE	80:20	Yes	94.9	92.4	95.8	95.2	96.4	92.2	95.6	96.4	94.7
KMeansSMOTE	70:30	Yes	93.6	92	95.7	92.5	99.2	90.6	94.9	91.8	98.2

## Results

### Datasets

Data for this research consists of 2 correlated secondary datasets, obtained through an open-source prostate cancer gene expression database, Xenabrowser GDC TCGA gene expression RNAseq Spliced Transcripts Alignment to a Reference–counts, and Xenabrowser GDC TCGA phenotypes. Gene expression RNAseq Spliced Transcripts Alignment to a Reference–counts contains 550 samples and 60,480 gene IDs in Ensembl format. On the other hand, the phenotype dataset contains 623 rows and 127 samples of clinical information on the samples included, from which sample types and race demographics columns are used to create a dataset based on race demographics. Out of the 550 samples present in the phenotype dataset, 458 were White, 12 were Asian, 1 was American Indian, 64 were African Americans, and 15 were not reported. The filtered-out White race count data that contains 57,429 gene IDs and 458 samples with their respective classes are presented in Multimedia Appendix 1 (Table S2).

### Feature Selection

To create a more enhanced feature selection method, several scenarios were made combining multiple methods based on DEG analysis thresholds. These scenarios reveal the most optimal combination of methods to identify genes relevant to prostate cancer.

From DEG analysis, various genes are extracted with several thresholds (Table S3 in Multimedia Appendix 1), the most being 139 genes. This result is further refined with ROC analysis and MSigDB investigation, which reveals 9 of 139 genes to have a direct correlation to prostate cancer.

Of the 139 genes identified through DEG analysis, PCA3 showed the strongest up-regulated correlation with prostate cancer (Table S4 in Multimedia Appendix 1). PCA3 had a baseMean of 12.33, indicating high expression across samples, a log2FoldChange of 0.6198, reflecting increased expression in cancerous tissue, and a *p-adj* value of <.001, confirming statistical significance.

Among the 139 genes identified from DEG analysis, WFDC2 has the strongest down-regulated correlation with prostate cancer (Table S5 in Multimedia Appendix 1). This is evident with a baseMean of 10.17 indicating a moderate expression level across samples, a log2FoldChange of −0.3069 which shows a decrease in expression levels in cancerous tissue compared to normal tissue, and a *p-adj*<.001 indicating high statistical significance after adjustment for multiple testing.

**Figure 2.**
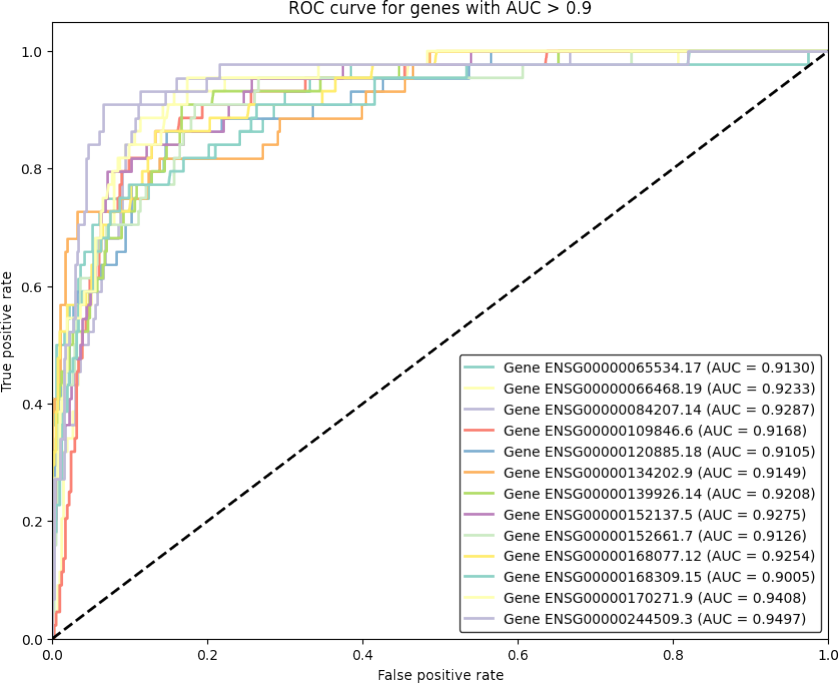
A total of 13 genes were identified to have a strong correlation (AUC>0.9) with prostate cancer obtained through ROC analysis of 139 genes. AUC: area under the curve; ROC: receiver operating characteristic.

ROC analysis was performed on 139 genes obtained using the White race DEG analysis, applying an area under the curve score threshold above 0.9. This process identified 13 genes as outliers, as shown in Figure 2, significantly narrowing down the initial gene set.

Genes that were identified from ROC analysis were converted from Ensembl format to gene symbol using BioTools.fr (Table S1 in Multimedia Appendix 1) to be verified through MSigDB.

**Figure 3.**
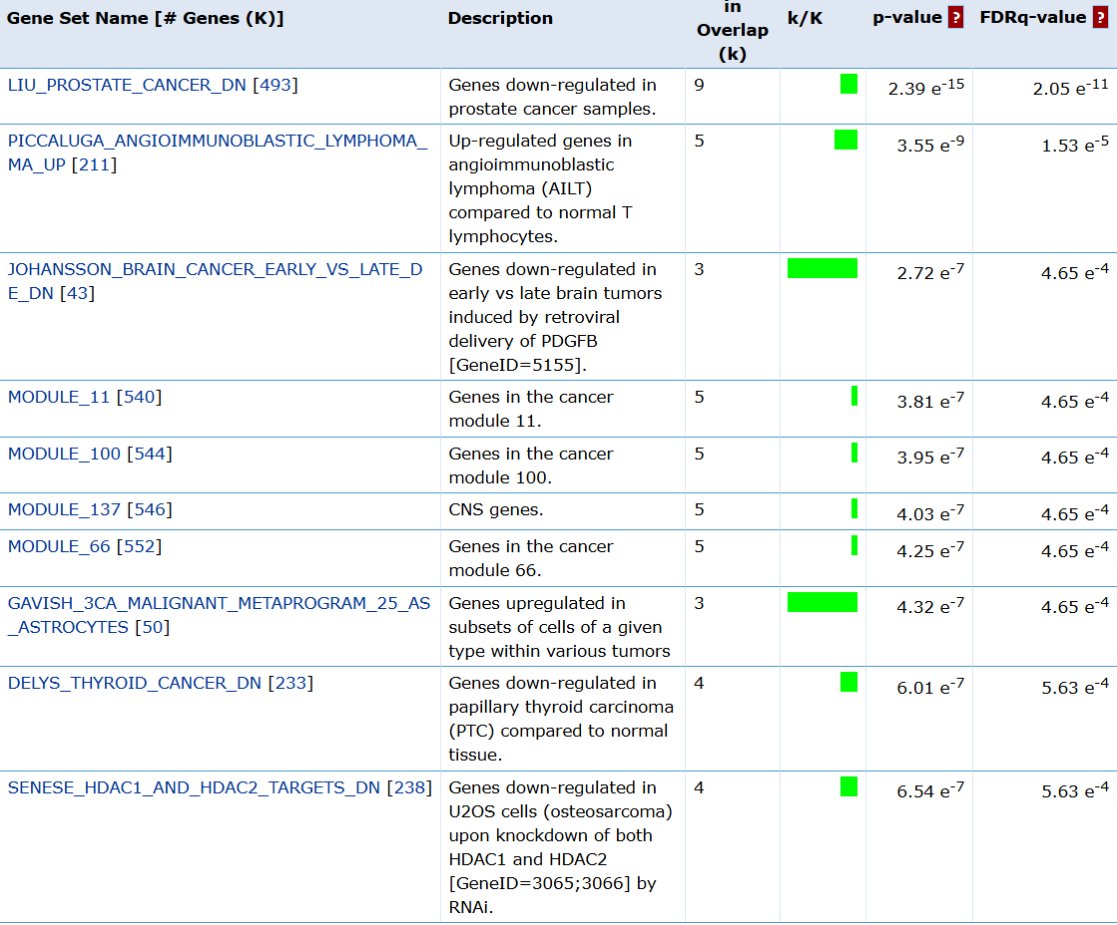
GSEA MSigDB investigation results of 139 genes selected from DEG analysis reveal 9 genes that are down-regulated in prostate cancer. 3CA / PIK3CA: phosphatidylinositol-4,5-bisphosphate 3-kinase catalytic subunit alpha; AILT: Angioimmunoblastic T-cell lymphoma; CNS: Central Nervous System; DEG: differentially expressed gene; FDR q: False Discovery Rate q-value; GSEA: gene set enrichment analysis; HDAC:Histone Deacetylase; k/K: is a ratio of number of genes in GSEA MSigDB data set (k) divided by the number of genes in the indicated dataset (K); LIU: protein LIU; MSigDB: Molecular Signature Database; PDGFB: Platelet-Derived Growth Factor Subunit B; PTC: papillary thyroid carcinoma; RNAi: RNA interference; U2OS: a human osteosarcoma cell line;

GSEA MSigDB investigation results reveal that the genes’ correlation varies between gene sets. We found that out of 13 genes, 9 were found to have a correlation to MSigDBs’ LIU_PROSTATE_CANCER_DN gene set with a *P*<.001 and False Discovery Rate q-value of 2.05 e^−11^ as seen in Figure 3.

### SVM Classifier

Various scenarios with different balancing methods and splitting percentages were implemented for constructing the ideal SVM model, creating minimal but important differences in class counts as seen in Multimedia Appendix 1 (Table S7).

From the various scenarios, we identified the top 5 best-performing models across different feature categories. The model using 139 genes from DEG analysis combined with the SMOTEENN balancing technique achieved the most consistent results, with a training accuracy of 100% and test accuracies of 97% for the White race and 96% for the Black race, alongside strong harmonization across *F*_1_-score, precision, and recall.

Compared to models using 4 and 7 genes, obtained through DEG analysis thresholds of log2FoldChange>0.35 and 0.4, achieved accuracies of 95% or below with unfavorable harmonization, thus the need for more advanced feature selection methods, such as ROC analysis combined with online GSEA. Models with 13 and 9 selected genes obtained through ROC analysis and GSEA demonstrated competitive performance, achieving 97% accuracy for the White race and 95% for the Black race, though slight deviations in precision and recall for the Black race were observed. Detailed metrics for all scenario models can be found from Tables 1-5.

**Table 2.** Top 5 models for 7-genes scenario.

Balancing method	Data splitting ratio	Hyper-parameter	White					Black			
			Train accuracy (%)	Test accuracy (%)	*F*_1_-score (%)	Precision (%)	Recall (%)	Test accuracy (%)	*F*_1_-score (%)	Precision (%)	Recall (%)
KMeansSMOTE	80:20	Yes	94.9	95.6	97.6	95.4	100	95.3	97.4	96.5	98.2
SVMSMOTE	80:20	Yes	97.9	94.6	97	96.4	97.6	90.6	94.6	96.4	93
KMeansSMOTE	80:20	No	94.4	94.6	97	95.3	98.8	93.7	96.5	94.9	98.2
KMeansSMOTE	60:40	No	96	92.9	96.1	94.7	97.6	95.3	97.4	96.5	98.2
SVMSMOTE	70:30	Yes	98.7	92.7	96.1	93.9	98.4	95.3	97.4	96.5	98.2

**Table 3.** Top 5 models for 9-genes scenario.

Balancing method	Data splitting ratio	Hyper-parameter	White					Black			
			Train accuracy (%)	Test accuracy (%)	*F*_1_-score (%)	Precision (%)	Recall (%)	Test accuracy (%)	*F*_1_-score (%)	Precision (%)	Recall (%)
SVMSMOTE	70:30	Yes	98.4	97.1	98.4	98.4	98.4	95.3	97.3	98.2	96.5
KMeansSMOTE	80:20	No	96.5	96.7	98.2	98.8	97.6	93.7	96.4	100	93
KMeansSMOTE	80:20	Yes	95.6	96.7	98.2	98.8	97.6	96.9	98.2	98.2	98.2
SMOTETomek	70:30	Yes	98.7	96.4	98	98.4	97.6	95.3	97.3	98.2	96.5
KMeansSMOTE	70:30	No	95.7	96.4	98	99.2	96.8	95.3	97.3	98.2	96.5

**Table 4.** Top 5 models for 13-genes scenario.

Balancing method	Data splitting ratio	Hyper-parameter	White					Black			
			Train accuracy (%)	Test accuracy (%)	*F*_1_-score (%)	Precision (%)	Recall (%)	Test accuracy (%)	*F*_1_-score (%)	Precision (%)	Recall (%)
KMeansSMOTE	70:30	No	95.2	97.1	98.4	99.2	97.6	95.3	97.3	98.2	96.5
SMOTETomek	80:20	Yes	98.1	96.7	98.2	97.6	98.8	95.3	97.3	100	94.7
BorderlineSMOTE	70:30	No	90.4	96.4	97.9	99.2	96.8	95.3	97.3	100	94.7
KMeansSMOTE	60:40	No	96.9	96.2	97.9	98.2	97.6	92.2	95.5	98.1	93
KMeansSMOTE	70:30	Yes	95.2	95.6	97.6	98.4	96.8	95.3	97.3	98.2	96.5

**Table 5.** Top 5 models for the 139 genes scenario.

Balancing method	Data splitting ratio	Hyper-parameter	White					Black			
			Train accuracy (%)	Test accuracy (%)	*F*_1_-score (%)	Precision (%)	Recall (%)	Test accuracy (%)	*F*_1_-score (%)	Precision (%)	Recall (%)
SMOTEENN	80:20	No	100	97.8	98.8	98.8	98.8	96.9	98.2	100	96.5
BorderlineSMOTE	60:40	Yes	98.8	97.3	98.5	99.4	97.6	96.9	98.2	100	96.5
SMOTEENN	70:30	Yes	100	97.1	98.4	99.2	97.6	96.9	98.2	100	96.5
SMOTEENN	70:30	No	100	97.1	98.4	99.2	97.6	96.9	98.2	100	96.5
SMOTEENN	80:20	Yes	100	96.7	98.2	98.8	97.6	96.9	98.2	100	96.5

## Discussion

### Principal Results

In this study, we explored multiple feature selection scenarios for race-based SVM classification models aimed at prostate cancer detection using gene expression data. Our findings demonstrate that race-based models with significantly reduced features are capable of achieving competitive performance comparable to models using thousands of genes. The best-performing model, achieved without hyperparameter tuning or cross-validation, demonstrated outstanding results with a training accuracy of 100% and test accuracies of 98% on the White race and 97% on the Black race. Additionally, the model showed strong harmonization across *F*_1_-score, precision, and recall values, which indicates consistent model classification performance. However, models in scenarios with 4 and 7 genes, selected using DEG analysis with thresholds of log2FoldChange>0.35 and 0.4, respectively, showed lower accuracies of 95% or lower, despite noteworthy harmonization between *F*_1_-score, precision, and recall values. This shows the limitations of feature selection solely using DEG analysis thresholds, as it failed to capture the critical biomarkers necessary for reliable classification.

Moreover, models with 9 and 13 selected genes through ROC analysis and GSEA present matched performance, achieving accuracies of 97% on the White race and 95% on the Black race. These models also demonstrated good stability, consistently performing well over different train-test dataset splits. While these reduced-feature models showed strong metrics for the White race, the slight drop in accuracy for the Black race indicates the presence of racial disparities in feature selection. This highlights the need for further research to improve model generalizability across more diverse populations.

### Strengths

This study addresses racial disparities in prostate cancer gene expression datasets to create a race-specific SVM classification model with multiple scenarios. Our testing demonstrated greater accuracies on scenarios using 139 genes; however, models with 13 and 9 selected genes also yielded 97% accuracy, highlighting the effectiveness of an optimized feature selection strategy. This feature reduction implies the significance of feature selection along with model construction parameters such as balancing methods, data splitting ratios, and hyperparameter optimization in achieving a robust classification model.

From a clinical standpoint, these results imply significant cost reduction and practical applicability. Reducing the number of genes required for sequencing substantially lowers the financial and computational cost of diagnostic workflows, making this approach more accessible and scalable for routine prostate cancer screening and early detection [47-49].

### Comparison With Prior Works

While prior works used feature selection methods with correlation-based and evolutionary algorithm approaches without further validations, our approach used tools such as *PyDESeq2* and MSigDB investigation to further validate the biological relevance of our selected genes to prostate cancer to improve the diagnostic accuracy and provide insights into race-specific prostate cancer biology, an area often neglected by other studies.

Our study achieved comparable accuracies to prior works while significantly reducing the number of features used. For example, Ravindran et al [29] reported a 97% accuracy while using 1833 features selected from the initial 6033 genes through a correlation-based approach [27]. Conversely, our models achieved similar accuracy using only 13 or 9 features, validating the performance of our feature selection method. Additionally, our study integrates racially based datasets to account for racial disparities while achieving robust performance for both the White (98% accuracy) and Black populations (97% accuracy). This further addresses the gap between prior works such as the model by Alshareef et al [27], with 52 prostate cancer samples and 1833 features, which overlook racial disparities [28]. To further appraise our model, we also compared it to a recent study by Xie and Xie [50] using an artificial neural network model on a DEG panel of 220 genes and reporting an accuracy of 78%, our optimized racial-based SVM model outperformed it with higher accuracy and fewer features, while maintaining consistent results across multiple dataset splits. These comparisons highlight the competitiveness and reliability of our SVM-based framework in prostate cancer detection.

### Limitations

However, this study has the following limitations. The datasets used are heavily imbalanced, with an overrepresentation of White individuals and cancer samples compared to normal samples. Only a single dataset source was used due to restricted access to other publicly available datasets, which limits the diversity and variability of the data. Future work should prioritize the inclusion of larger, more diverse populations to enhance the model’s generalizability and consider an external independent dataset to validate the model’s performance. Additionally, exploring other genomic and epigenomic features, such as DNA methylation patterns, may yield further insights into race-specific prostate cancer biology.

### Conclusions

This research used enhanced feature selection methods such as DESeq2 DEG analysis and ROC analysis to reduce feature quantity in machine learning models for prostate cancer detection in specific racial groups. Our findings show that while testing on White race reducing features-maintained model, performance was comparable to studies with larger feature sets. To examine racial disparities, we tested the model on African American data, revealing minimal (~1%) accuracy differences between racial groups. These findings indicate a low influence of racial features on classification while emphasizing the importance of feature selection in developing race-based SVM models for prostate cancer using gene expression data.

## Supplementary material

10.2196/72423 Multimedia Appendix 1 Tables on modeling scenarios, dataset, genes, and the machine learning training model.

## Abbreviations

DEG: differentially expressed gene
GDC: Genomic Data Commons
GSEA: gene set enrichment analysis
MGECD: Micro Gene Expression Cancer Dataset
MSigDB: Molecular Signature Database
PSA: prostate-specific antigen
ROC: receiver operating characteristic
SVM: support vector machine
TCGA: The Cancer Genome Atlas
TCGA-PRAD: The Cancer Genome Atlas Prostate Adenocarcinoma
