# Supplementary material for: Framework for Race-Specific Prostate Cancer Detection Using Machine Learning Through Gene Expression Data: Feature Selection Optimization Approach
Source: JMIR Bioinform Biotechnol. 2025 Jul 31;6:e72423. doi: 10.2196/72423 (PMC12314727; doi:10.2196/72423)
Supplement: Multimedia Appendix 1 [file bioinform-v6-e72423-s001.doc]

**Table S1.** Modeling scenarios based on the number of features obtained from feature selection.

| **Feature selection method** | **Data splitting ratio** | **Training data balancing ratio and technique** | **Hyperparameter tuning** |
| --- | --- | --- | --- |
| DEG^a^ with basemean >=10 & p-adj < .05 | 60:40, 70:30, 80:20 | No balancing or ROS^b^, KMeansSMOTE, SVMSMOTE, SMOTEENN, BorderlineSMOTE | Yes or no |
| DEG with basemean >= 10 & p-adj < .05 & abs(log2FoldChange) > 0.35 | 60:40, 70:30, 80:20 | No balancing or ROS, KMeansSMOTE, SVMSMOTE, SMOTEENN, BorderlineSMOTE | Yes or no |
| DEG with basemean >= 10 & p-adj < .05 & abs(log2FoldChange) > 0.4 | 60:40, 70:30, 80:20 | No balancing or ROS, KMeansSMOTE, SVMSMOTE, SMOTEENN, BorderlineSMOTE | Yes or no |
| ROC^c^ analysis of 139 genes from DEG | 60:40, 70:30, 80:20 | No balancing or ROS, KMeansSMOTE, SVMSMOTE, SMOTEENN, BorderlineSMOTE | Yes or no |
| ROC analysis of 139 genes from DEG and MSigDB^d^ Investigation | 60:40, 70:30, 80:20 | No balancing or ROS, KMeansSMOTE, SVMSMOTE, SMOTEENN, BorderlineSMOTE | Yes or no |

**Notes:** ^a^DEG: differentially expressed gene.^b^ROS: RandomOverSampler.^c^ROC: receiver operating characteristic.^d^MSigDB: Molecular Signature Database.

**Table S2.** Dataset sample counts per race.

| **Race** | **Total sample** | **Cancer** | **Normal** |
| --- | --- | --- | --- |
| White | 458 | 414 | 44 |
| African American | 64 | 57 | 7 |
| American Indian | 1 | 1 | 0 |
| Asian | 12 | 12 | 0 |
| Not Reported | 15 | 14 | 1 |
| Total | 550 | 498 | 52 |

**Table S3.** Feature selection scenarios and the number of genes extracted.

| **Feature selection method** | **Identified genes** |
| --- | --- |
| DEG^a^ with basemean >=10 & p-adj < .05 | 139 |
| ROC^b^ analysis of 139 genes from DEG | 13 |
| ROC analysis of 139 genes from DEG and GSEA^c^ MSigDB^d^ Investigation | 9 |
| DEG with basemean >= 10 & p-adj < .05 & abs(log2FoldChange) > 0.35 | 7 |
| DEG with basemean >= 10 & p-adj < .05 & abs(log2FoldChange) > 0.4 | 4 |

**Notes:** ^a^DEG: differentially expressed gene.^b^ROC: receiver operating characteristic.^c^GSEA: gene set enrichment analysis.^d^MSigDB: Molecular Signature Database.

**Table S4.** Top 10 up-regulated outlier genes from DEG analysis with baseMean ≥10 and p <.05.

| **Ensembl_ID** | **Gene symbol** | **baseMean** | **log2FoldChange** | **p-adj** |
| --- | --- | --- | --- | --- |
| ENSG00000225937.1 | PCA3 | 12.3377 | 0.6198 | <.001 |
| ENSG00000242899.1 | RPL7P16 | 11.2315 | 0.4366 | <.001 |
| ENSG00000105664.9 | COMP | 10.109 | 0.4045 | <.001 |
| ENSG00000166743.8 | ACSM1 | 10.7674 | 0.4001 | <.001 |
| ENSG00000242110.6 | AMACR | 11.8239 | 0.3885 | <.001 |
| ENSG00000159263.14 | SIM2 | 11.5686 | 0.3761 | <.001 |
| ENSG00000280623.1 | PCAT14 | 12.6492 | 0.3736 | <.001 |
| ENSG00000138028.13 | CGREF1 | 10.278 | 0.3471 | <.001 |
| ENSG00000187398.10 | LUZP2 | 10.0539 | 0.3321 | <.001 |
| ENSG00000167332.7 | OR51E2 | 13.5529 | 0.327 | <.001 |

**Notes:** ^a^DEG: differentially expressed gene.

**Table S5.** Top 10 down-regulated outlier genes from DEG analysis with baseMean>=10 and p-adj <.05.

| **Ensembl_ID** | **Gene symbol** | **baseMean** | **log2FoldChange** | **p-adj** |
| --- | --- | --- | --- | --- |
| ENSG00000101443.16 | WFDC2 | 10.1703 | −0.3069 | <.001 |
| ENSG00000244509.3 | APOBEC3C | 10.0521 | −0.3012 | <.001 |
| ENSG00000196878.11 | LAMB3 | 10.5885 | −0.2776 | <.001 |
| ENSG00000004468.11 | CD38 | 10.094 | −0.2707 | <.001 |
| ENSG00000186081.10 | KRT5 | 11.9698 | −0.2683 | <.001 |
| ENSG00000066468.19 | FGFR2 | 10.1722 | −0.2635 | <.001 |
| ENSG00000137699.15 | TRIM29 | 10.8605 | −0.2618 | <.001 |
| ENSG00000102837.6 | OLFM4 | 10.1606 | −0.25 | <.001 |
| ENSG00000101938.13 | CHRDL1 | 11.2763 | −0.2489 | <.001 |
| ENSG00000141469.15 | SLC14A1 | 10.5671 | −0.2485 | <.001 |

**Notes:** ^a^DEG: differentially expressed gene.

**Table S6.** A total of 13 Ensembl IDs obtained from ROC^a^ analysis and their respective gene symbol.

| **Ensembl_ID** | **Gene symbol** |
| --- | --- |
| ENSG00000065534.17 | MYLK |
| ENSG00000066468.19 | FGFR2 |
| ENSG00000084207.14 | GSTP1 |
| ENSG00000109846.6 | CRYAB |
| ENSG00000120885.18 | CLU |
| ENSG00000134202.9 | GSTM3 |
| ENSG00000139926.14 | FRMD6 |
| ENSG00000152137.5 | HSPB8 |
| ENSG00000152661.7 | GJA1 |
| ENSG00000168077.12 | SCARA3 |
| ENSG00000168309.15 | FAM107A |
| ENSG00000170271.9 | FAXDC2 |
| ENSG00000244509.3 | APOBEC3C |

**Notes:** ^a^ROC: receiver operating characteristic.

**Table S7.** Balancing samples class (cancer and normal) for the machine learning training model.

| **Balancing method and splitting percentage (train/test)** | | **Number of training datasets before balancing** | | **Number of training datasets after balancing** | |
| --- | --- | --- | --- | --- | --- |
|  | | **Cancer** | **Normal** | **Cancer** | **Normal** |
| SMOTEENN | |  |  |  |  |
|  | 80%/20% | 331 | 35 | 294 | 94 |
|  | 70%/30% | 289 | 31 | 260 | 75 |
|  | 60%/40% | 274 | 26 | 220 | 68 |
| BorderlineSMOTE | |  |  |  |  |
|  | 80%/20% | 331 | 35 | 331 | 99 |
|  | 70%/30% | 289 | 31 | 289 | 86 |
|  | 60%/40% | 274 | 26 | 248 | 74 |
| SMOTETomek | |  |  |  |  |
|  | 80%/20% | 331 | 35 | 330 | 98 |
|  | 70%/30% | 289 | 26 | 289 | 86 |
|  | 60%/40% | 274 | 26 | 248 | 74 |
| RandomOverSampler | |  |  |  |  |
|  | 80%/20% | 331 | 35 | 331 | 99 |
|  | 70%/30% | 289 | 31 | 289 | 86 |
|  | 60%/40% | 274 | 26 | 248 | 74 |
| SVMSMOTE | |  |  |  |  |
|  | 80%/20% | 331 | 35 | 331 | 99 |
|  | 70%/30% | 289 | 31 | 289 | 86 |
|  | 60%/40% | 274 | 26 | 248 | 74 |
| KMeansSMOTE | |  |  |  |  |
|  | 80%/20% | 331 | 35 | 331 | 99 |
|  | 70%/30% | 289 | 31 | 289 | 86 |
|  | 60%/40% | 274 | 26 | 248 | 75 |
| ADASYN | |  |  |  |  |
|  | 80%/20% | 331 | 35 | 331 | 102 |
|  | 70%/30% | 289 | 31 | 289 | 86 |
|  | 60%/40% | 274 | 26 | 248 | 74 |
